# Colchicine improves clinical outcomes in patients with coronary disease, will it result in similar benefits in peripheral artery disease?

**DOI:** 10.1093/ehjopen/oeae063

**Published:** 2024-08-06

**Authors:** Jean-Claude Tardif, Sabine Cuthill

**Affiliations:** Department of Medicine, Montreal Heart Institute, 5000 Belanger Street, Montreal, PQ, Canada H1T 1C8; Department of Medicine, Montreal Heart Institute, 5000 Belanger Street, Montreal, PQ, Canada H1T 1C8

**This editorial refers to ‘Colchicine for cardiovascular and limb risk reduction in Medicare beneficiaries with peripheral artery disease: emulation of target trials’, by P. Heindel *et al.,*  https://doi.org/10.1093/ehjopen/oeae062.**

Inflammation is central to the initiation, progression, and destabilization of atherosclerosis. The anti-inflammatory agent colchicine has been shown to reduce the risk of vascular events both in patients with a recent myocardial infarction and in those with chronic coronary artery disease.^1,2^ This has led to the introduction of low-dose colchicine in European and American prevention guidelines.^3,4^ Furthermore, the use of colchicine has been approved by the US Food and Drug Administration to reduce the risk of myocardial infarction, stroke, coronary revascularization, and cardiovascular death in adult patients with established atherosclerotic disease. Whether colchicine will yield similar benefits in patients with peripheral vascular disease is presently not known.

In this issue of the European Heart Journal Open, Heindel *et al.* attempt to evaluate the effects of colchicine in patients with peripheral artery disease.^5^ Because no randomized placebo-controlled trial addressing this question has been completed, the authors simulated two clinical studies of patients with peripheral artery disease and gout treated with a urate-lowering medication (predominantly allopurinol). The first simulated study compared the initiation of treatment with colchicine or with a variety of non-steroidal anti-inflammatory drugs. The second simulated study compared ‘long-term’ and short-term (3 months) treatment with colchicine. Patients were eligible if they were continuously enrolled in Medicare and receiving care at an American academic center between 2007 and 2019. These simulated studies failed to demonstrate significant differences between treatment arms in clinical endpoints pertaining to cardiovascular and limb events.

Although Heindel *et al.* used the best possible approach in their analysis, these ‘emulated trials’ suffer from major limitations. First, these two non-randomized studies were underpowered and included a total of 1820 patients, in contrast to the COLCOT and LoDoCo2 randomized trials that included collectively more than 10 000 patients. Second, the mean therapy duration was exceedingly short at 247 days for colchicine and 137 days for non-steroidal anti-inflammatory drugs. Such brief treatment periods do not allow for any credible conclusion on the effects on clinical outcomes in patients with chronic atherosclerotic disease. Third, the use of concomitant medications was lower than expected, with statins and prescription anti-platelet agents used in 77% and 14% of patients, respectively. Fourth, non-randomized studies cannot necessarily account for all potential confounders and ensure that all baseline characteristics are similar among treatment groups. Multiple factors likely were taken into consideration before initiating colchicine or a non-steroidal anti-inflammatory drug in these patients who were not randomly assigned to treatment. In the analysis of the subset of 989 patients with coronary artery disease, the emulated trials could not replicate the benefits on cardiovascular outcomes observed in the COLCOT and LoDoCo2 randomized trials. This appears to put in question the other results of the simulated studies reported here by Heindel *et al.*

The COLCOT and LoDoCo2 randomized trials have shown that low-dose colchicine reduces the risk of cardiovascular events in patients with established coronary artery disease. Ongoing randomized clinical trials like LEADER-PAD (NCT04774159) and COLCOT-T2D (NCT05633810) will inform us on the effects of colchicine in other types of patients like those living with peripheral artery disease and Type 2 diabetes.

## Data Availability

The data underlying this article will be shared on reasonable request to the corresponding author.
